# *Lactobacillus rhamnosus* attenuates bone loss and maintains bone health by skewing Treg-Th17 cell balance in Ovx mice

**DOI:** 10.1038/s41598-020-80536-2

**Published:** 2021-01-19

**Authors:** Leena Sapra, Hamid Y. Dar, Asha Bhardwaj, Amit Pandey, Surbhi Kumari, Zaffar Azam, Vishu Upmanyu, Aleena Anwar, Prashant Shukla, Pradyumna K. Mishra, Chaman Saini, Bhupendra Verma, Rupesh K. Srivastava

**Affiliations:** 1 Department of Biotechnology, All India Institute of Medical Sciences (AIIMS), New Delhi, 110029 India; 2 Division of Endocrinology, School of Medicine, Emory University, Atlanta, GA 30322 USA; 3 Department of Physics, Dr. Harisingh Gour Central University, Sagar, MP 470003 India; 4 Department of Molecular Biology, ICMR-National Institute for Research in Environmental Health, Bhopal, MP 462001 India; 5 Department of Zoology, Dr. Harisingh Gour Central University, Sagar, MP 470003 India

**Keywords:** Osteoimmunology, Endocrinology

## Abstract

Osteoporosis is a systemic-skeletal disorder characterized by enhanced fragility of bones leading to increased rates of fractures and morbidity in large number of populations. Probiotics are known to be involved in management of various-inflammatory diseases including osteoporosis. But no study till date had delineated the immunomodulatory potential of *Lactobacillus rhamnosus* (LR) in bone-health. In the present study, we examined the effect of probiotic-LR on bone-health in ovariectomy (Ovx) induced postmenopausal mice model. In the present study, we for the first time report that LR inhibits osteoclastogenesis and modulates differentiation of Treg-Th17 cells under in vitro conditions. We further observed that LR attenuates bone loss under in vivo conditions in Ovx mice. Both the cortical and trabecular bone-content of Ovx+LR treated group was significantly higher than Ovx-group. Remarkably, the percentage of osteoclastogenic CD4^+^Rorγt^+^Th17 cells at distinct immunological sites such as BM, spleen, LN and PP were significantly reduced, whereas the percentage of anti-osteoclastogenic CD4^+^Foxp3^+^Tregs and CD8^+^Foxp3^+^Tregs were significantly enhanced in LR-treated group thereby resulting in inhibition of bone loss. The osteoprotective role of LR was further supported by serum cytokine data with a significant reduction in osteoclastogenic cytokines (IL-6, IL-17 and TNF-α) along with enhancement in anti-osteoclastogenic cytokines (IL-4, IL-10, IFN-γ) in LR treated-group. Altogether, the present study for the first time establishes the osteoprotective role of LR on bone health, thus highlighting the immunomodulatory potential of LR in the treatment and management of various bone related diseases including osteoporosis.

## Introduction

Bone is a dynamic organ that maintains its proper architecture and function by undergoing continuous cycles of modelling and remodelling and thus, helps in maintaining normal host physiology. Any dysregulation in the bone remodelling process results in development of bone related diseases including osteoporosis. Patients diagnosed with osteoporosis exhibit low bone mineral density along with compromised bone microarchitecture resulting in higher risk of fractures^1–4^. Among the global population, greater prevalence of osteoporosis has been observed in postmenopausal women due to lower estrogen levels^5^. Various FDA approved drugs and monoclonal antibodies are being currently used for treatment of osteoporosis, but unfortunately all these compounds along with providing relief to the patients also result in various side effects^6^. Due to increase in ageing population across the globe, osteoporosis is now becoming a budding medical and socioeconomic issue worldwide and thus there is an exigent need to identify safer and cost-effective interventions exhibiting both preventative and therapeutic abilities for management of osteoporosis.

For past few years, Treg and Th17 cells have gained tremendous attention due to their association with various autoimmune and inflammatory diseases^7^. CD4^+^Foxp3^+^Treg cells via secreting anti-inflammatory or immunosuppressive cytokines such as IL-10 and TGF-β suppress osteoclastogenesis and bone resorption in a cytokine dependent manner^3,8^. On the contrary, CD4^+^Rorγt^+^Th17 cells via secreting IL-6, IL-17 and TNF-α inflammatory cytokines enhance the expression of RANKL on osteoblasts and fibroblasts and thus promotes osteoclast mediated bone resorption^9^. RANKL is a crucial cytokine involved in differentiation of osteoclasts precursors and in survival of mature osteoclasts. RANKL is expressed by variety of cells such as osteoblasts, T cells and B cells^10^. Furthermore, several studies demonstrated that decline in estrogen levels not only effects the bone forming (osteoblastogenesis) and bone resorbing (osteoclastogenesis) process but is also known to affect functionality of immune cells especially T cells^10–12^. The homeostatic balance of Treg-Th17 cell axis is an important determinant of enhanced bone-loss in osteoporosis^13,14^. Estrogen exhibits the potential to stimulate the differentiation and survival of Tregs which has already been shown to suppress bone resorption^15^. Tyagi et al. demonstrated that estrogen via suppressing secretion of inflammatory cytokines from Th17 cells suppresses bone resorption^16^. Thus, bone-loss associated with estrogen deficiency may occur due to the impairment in complex network of hormones and cytokines that interrupts the bone remodelling process. In lieu of the growing involvement of immune system in osteoporosis, our group has recently coined the term “Immunoporosis” (i.e. Immunology of Osteoporosis) to highlight the specific role of immune system in osteoporosis^3^.

In recent years, “gut-bone” axis has gained tremendous attention. It has been observed that any dysbiosis in the intestinal microbiota leads to pathogenesis of various diseases such as IBD, obesity, diabetes, RA etc. which can further lead to bone-loss and development of secondary osteoporosis^17–21^. The appreciation that bacterial species can impart numerous benefits to human health dates to ancient times. World health organization (WHO) defines probiotics as “viable microorganisms acting as nutritional supplement that confers various health benefits when administered in adequate amounts”^22,23^. Also, several animal and small scale human studies reported that intake of probiotics showed positive results in both Ovx mice and osteoporotic patients^13,14,24,25^. Recently our group too have reported the osteoprotective properties of both *Lactobacillus acidophilus* (LA) and *Bacillus clausii* (BC) probiotics in Ovx mice^13,14^.

One of the widely studied probiotic strain of *Lactobacillus* species for various human applications is *Lactobacillus rhamnosus* (LR). It is a gram positive, anaerobic bacterium that exhibits the capacity to transport and metabolize carbohydrates and thereby helps in maintaining the epithelial-layer gut integrity^26^. Recently, a study demonstrated that LR-administration alleviates gut inflammation and improved barrier function of intestine^27^. But the immunoregulatory role of LR in regulating bone-health is still required. Thus, in the present study we aim to investigate the immunoregulatory role of LR on bone health in Ovx mice.

Herein, we report for the first time that LR inhibits osteoclastogenesis and skews balance of Treg-Th17 cells under in vitro conditions. Thus, administration of LR suppresses bone resorption and maintains bone mass in Ovx mice by maintaining the balance between Treg-Th17 cells equilibrium in bone marrow (BM), peyer’s patch (PP), spleen and lymph nodes (LN). The immunomodulatory potential of LR was further supported by our in vivo serum cytokine data, in which we found augmented levels of anti-osteoclastogenic cytokines (IL-4, IL-10 and IFNγ) along with simultaneous suppression of osteoclastogenic cytokines/factors (IL-6, IL-17, TNF-α and RANKL). Collectively, the present study highlights the osteoprotective role of LR, thereby opening novel avenues in the management and treatment of postmenopausal osteoporosis.

## Results

### *Lactobacillus rhamnosus* (LR) inhibits osteoclastogenesis in vitro

**Figure 1:**
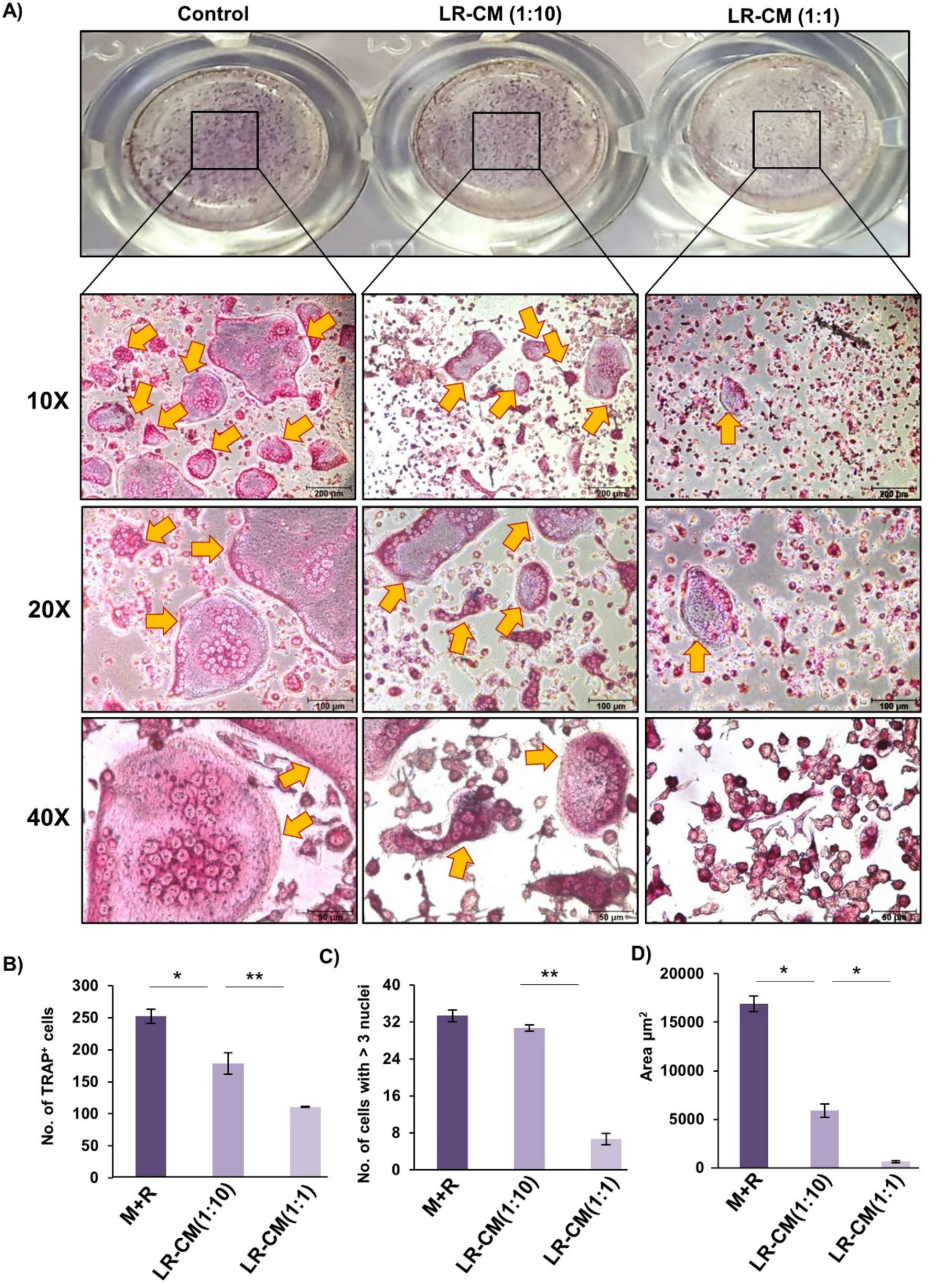
LR-CM inhibits osteoclastogenesis in a dose dependent manner: Osteoclasts differentiation was induced in Bone Marrow Macrophages (BMMs) with M-CSF (30 ng/ml) and RANKL (100 ng/ml) with or without *Lactobacillus rhamnosus*—conditioned media (LR-CM) at different ratios of 1:10 and 1:1 for 6 days. Giant multinucleated cells were stained with TRAP and cells with ≥ 3 nuclei were considered as mature osteoclasts. (**A**) Photomicrographs at different magnifications (10×, 20× and 40×) were taken. (**B**) Number of TRAP positive cells. (**C**) Number of TRAP positive cells with more than 3 nuclei. (**D**) Area of osteoclasts. The above images are indicative of one independent experiment and similar results were obtained in three different independent experiments. Statistical significance was considered as *p* ≤ 0.05 (*p ≤ 0.05, **p ≤ 0.01, ***p ≤ 0.001) with respect to indicated groups.

To determine whether LR possesses potential to modulate bone health, we first examined the effect of LR on RANKL induced osteoclasts differentiation under in vitro conditions. In order to study the same, we prepared LR-conditioned media (LR-CM) by culturing LR in α-MEM media for 3 h and the supernatant was collected and further used as LR-CM. For in vitro osteoclastogenesis assay, mice bone marrow macrophages were stimulated with M-CSF (30 ng/ml) and RANKL (100 ng/ml) in the presence or absence of LR-CM at different ratios (viz. 1:10 and 1:1). After five days, cells were fixed and stained for Tartrate resistant acid phosphatase (TRAP) to identify differentiated multinucleated osteoclasts. Interestingly, we observed that LR-CM treatment significantly decreased the osteoclasts differentiation in a dose dependent manner estimated by the significantly reduced TRAP positive cells in LR-CM treated groups in comparison to control group (Fig. 1A). Furthermore, area measurement analysis of multinucleated TRAP positive cells using Image J software revealed significant reduction (25-fold) in the area of TRAP positive osteoclasts in the treatment groups (Fig. 1B–D). To exclude the possibility that the observed reduction in osteoclasts differentiation and number is not due to cell cytotoxicity, MTT assay was performed and we found no significant difference in cell viability with LR-CM treatment at different dilutions (data not shown). Thus, our data clearly suggest that LR has potential to inhibit RANKL induced osteoclastogenesis under in vitro conditions*.*

### LR inhibits F-actin ring formation

**Figure 2:**
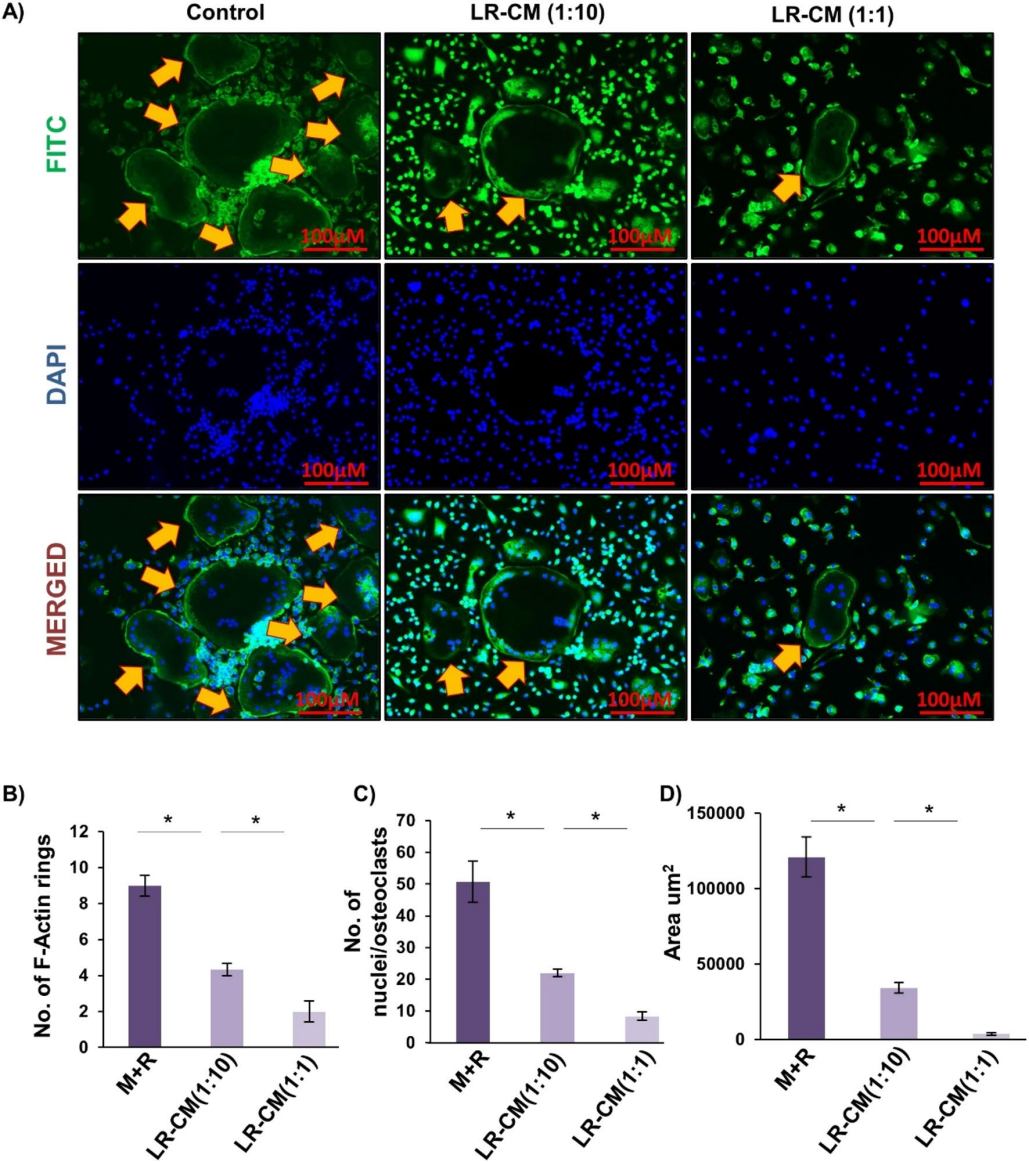
LR-CM inhibits RANKL stimulated F-actin ring formation: Bone Marrow Macrophages (BMMs) were treated with M-CSF (30 ng/ml) and RANKL (100 ng/ml) with or without *Lactobacillus rhamnosus*—Conditioned Media (LR-CM) at different ratios of 1:10 and 1:1 for 6 days. F-actin and nuclei were stained with FITC-conjugated phalloidin and DAPI respectively. Images were captured in fluorescence microscope (Imager.Z2 Zeiss microscope) at 10× magnification. (**B**) Number of F-actin rings. (**C**) Number of nuclei per osteoclasts. (**D**) Area of F-actin rings. The above images are indicative of one independent experiment and similar results were obtained in three different independent experiments. Statistical significance was considered as *p* ≤ 0.05 (*p ≤ 0.05, **p ≤ 0.01, ***p ≤ 0.001) with respect to indicated groups.

F-actin ring formation is a visual phenotype of mature osteoclasts for mediating its functional activity i.e. bone resorption^28^. Thus, we next addressed the effect of LR on F-actin ring formation by fixing and permeabilizing bone marrow derived osteoclasts on glass coverslips and further cells were stained for F-actin (FITC-labelled phalloidin) and nuclei (DAPI) respectively. Strikingly, we observed that with respect to control group, LR-CM treatment in a dose dependent manner significantly decreased the number and area of F-actin rings (Fig. 2A–D). Consistent with our previous results these data further confirm the role of LR in inhibiting osteoclastogenesis in vitro.

### LR attenuates bone loss in ovariectomized mice

**Figure 3:**
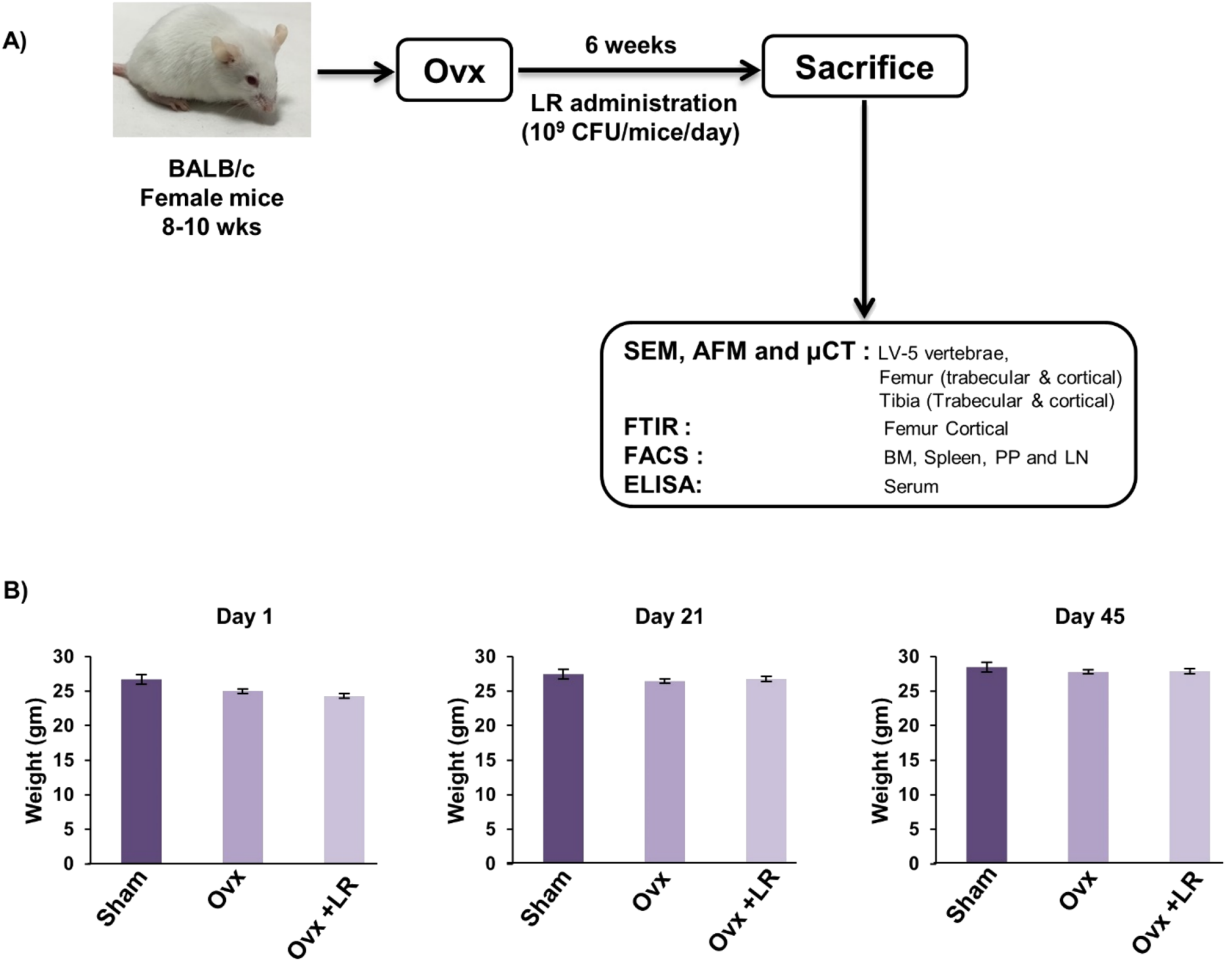
Experimental work plan for in vivo experiments. (**A**) Mice were divided into 3 groups. viz. Sham group, Ovx and Ovx+LR received LR. At the end of experiment (D45), mice were sacrificed and further studies performed (Mouse Image courtesy: Leena Sapra). (**B**) Effect of *Lactobacillus rhamnosus* (LR) on body weight; values are reported as mean ± SEM (n = 6/gp).

**Figure 4:**
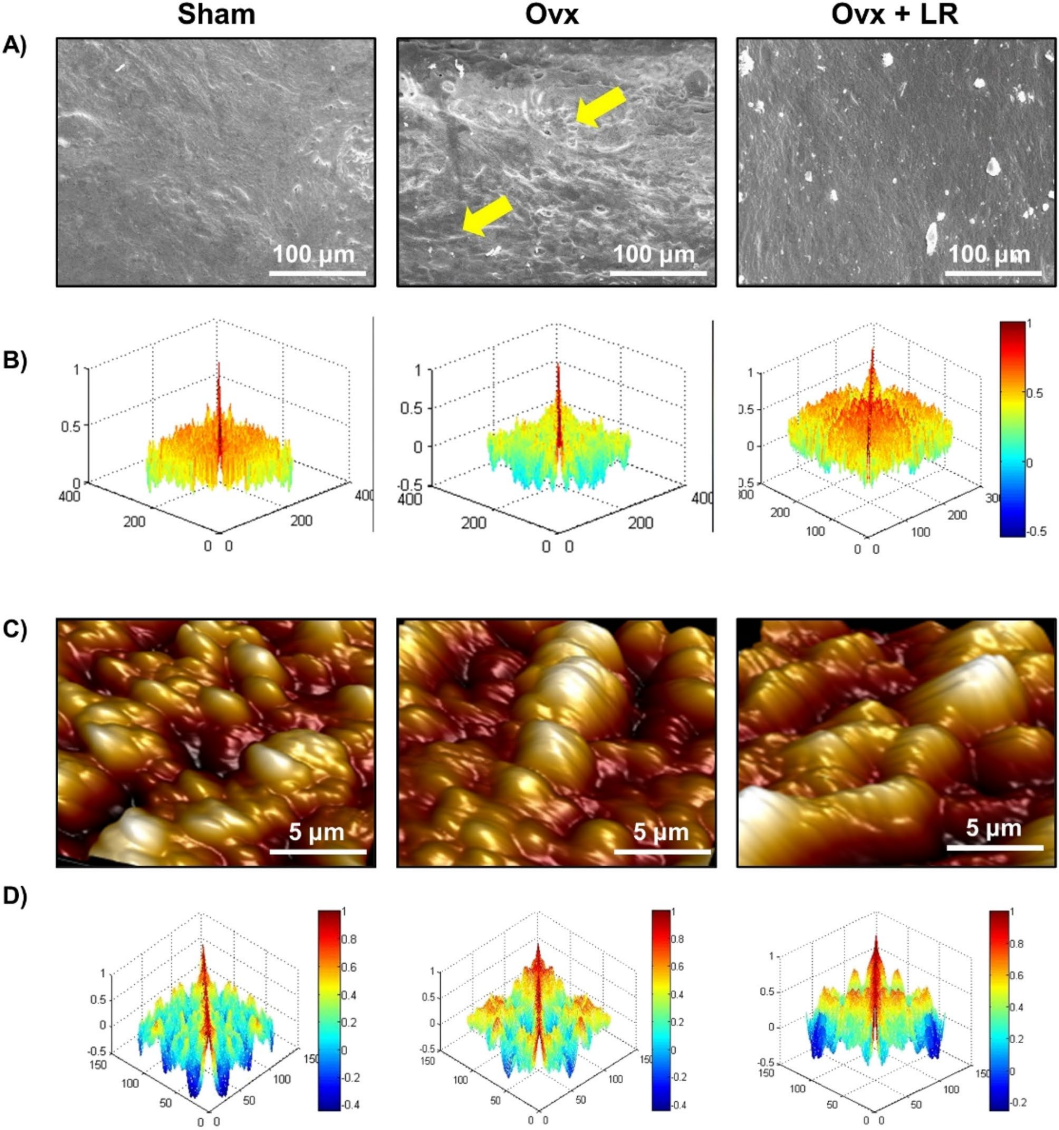
LR attenuates bone loss in Ovx mice. Mice were sacrificed at the end of experiment and cortical bones of all groups were collected for SEM and AFM analysis. (**A**) 2D SEM images. (**B**) 2D MATLAB analysis of SEM images. (**C**) 3D AFM images. (**D**) 2D MATLAB analysis of AFM image. The above images are indicative of one independent experiment and comparable results were obtained in two different independent experiments with n = 6 mice/group/experiment.

To test whether these in vitro findings of LR could be exploited as a novel strategy in the treatment of pathological bone loss conditions, we next investigated the role of LR in modulating bone health in Ovx-induced postmenopausal osteoporotic mice model. For accomplishing the same, female BALB/c mice were divided in three groups viz. Sham, Ovx and Ovx+LR and after one-week post-surgery, LR was administered orally for a period of 6wks. At the end of experiment, mice were sacrificed, and bones were harvested for further analysis (Fig. 3A). During dosage period, we also examined body weight of all groups at regular intervals (Day 1, 21 and 45) but no difference in weight was observed in Ovx and LR treated group (Fig. 3B). To investigate whether LR-administration specifically inhibits bone-loss in Ovx mice, we further studied the effect of LR-administration on bone pathology and bone remodelling processes. Scanning electron microscopy (SEM) analysis of cortical region of femoral bones revealed that mice of Ovx group had enhanced number of resorption pits or lacunae representing higher osteoclastogenesis (Fig. 4A). Strikingly, LR administration significantly reduced the resorption pits/lacunae in femoral bones of Ovx group, a clear sign of reduced/inhibited osteoclastogenesis (Fig. 4A). To further analyse SEM images quantitatively in a more statistical manner, we employed MATLAB (matrix-laboratory) to derive the correlation between bone mass and bone loss. MATLAB analysis of 2D-SEM images signifies the degree of homogeneity where red colour symbolizes higher correlation (high bone mass) whereas blue colour symbolizes lesser correlation (more bone loss). MATLAB-data of SEM (Fig. 4B), clearly points that Ovx+LR group has greater correlation and thus more bone mass. Since SEM images illustrate 2D-information of bone samples and we were also interested to study the 3D-topology of bone-structures, so we next performed atomic force microscopy (AFM) analysis of cortical region of femoral bone. Our AFM data showed a significant suppression of bone resorption in Ovx+LR administered group in comparison to Ovx group (Fig. 4C). Moving ahead, MATLAB-analysis of AFM-3D images (Fig. 4D) was performed in which red colour represents enhanced bone architecture (reduced osteoclastogenesis) and blue colour representing reduced bone architecture (enhanced osteoclastogenesis). This AFM data thus further supplement and validate our earlier SEM data and support our hypothesis that LR inhibits bone loss in Ovx mice. Taken together, our findings from both SEM and AFM data suggest that LR treatment prevents bone loss in Ovx mice.

### LR enhances bone microarchitecture in ovariectomized mice

**Figure 5:**
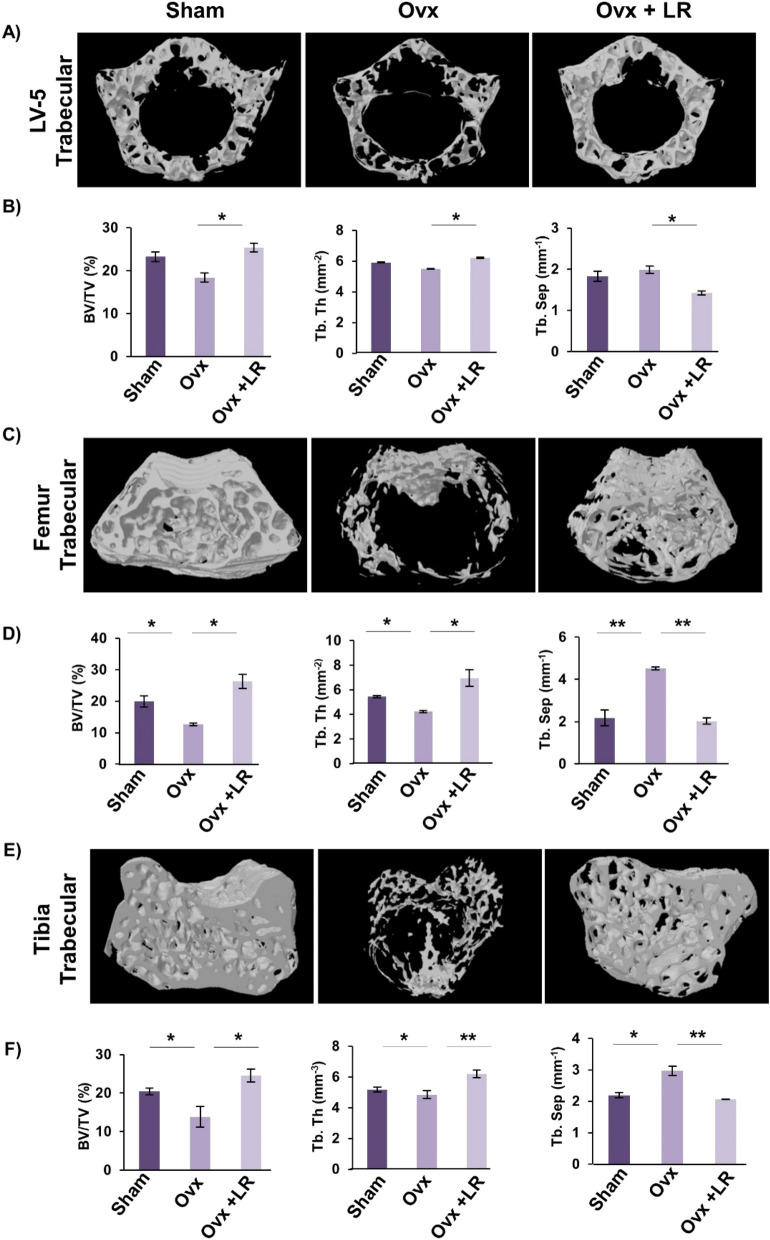
LR administration enhances trabecular bone microarchitecture. 3-D µCT reconstruction of LV-5 Trabecular, Femur Trabecular and Tibia Trabecular of all groups. (**A**) Bone micro-architecture of LV-5. (**B**) Histomorphometric parameters of LV-5. (**C**) Bone micro-architecture of femur trabecular. (**D**) Histomorphometric parameters of femur trabecular. (**E**) Bone micro-architecture of Tibia trabecular. (**F**) Histomorphometric parameters of Tibia trabecular. Bone volume/tissue volume ratio (BV/TV); Tb. Th., trabecular thickness; Tb. Sp., trabecular separation. The results were evaluated by ANOVA with subsequent comparisons by Student t-test for paired or nonpaired data. Values are reported as mean ± SEM. The above graphical representations are indicative of one independent experiment and similar results were obtained in two different independent experiments with n = 6. Statistical significance was considered as *p* ≤ 0.05 (*p ≤ 0.05, **p ≤ 0.01, ***p ≤ 0.001) with respect to indicated mice groups.

**Figure 6:**
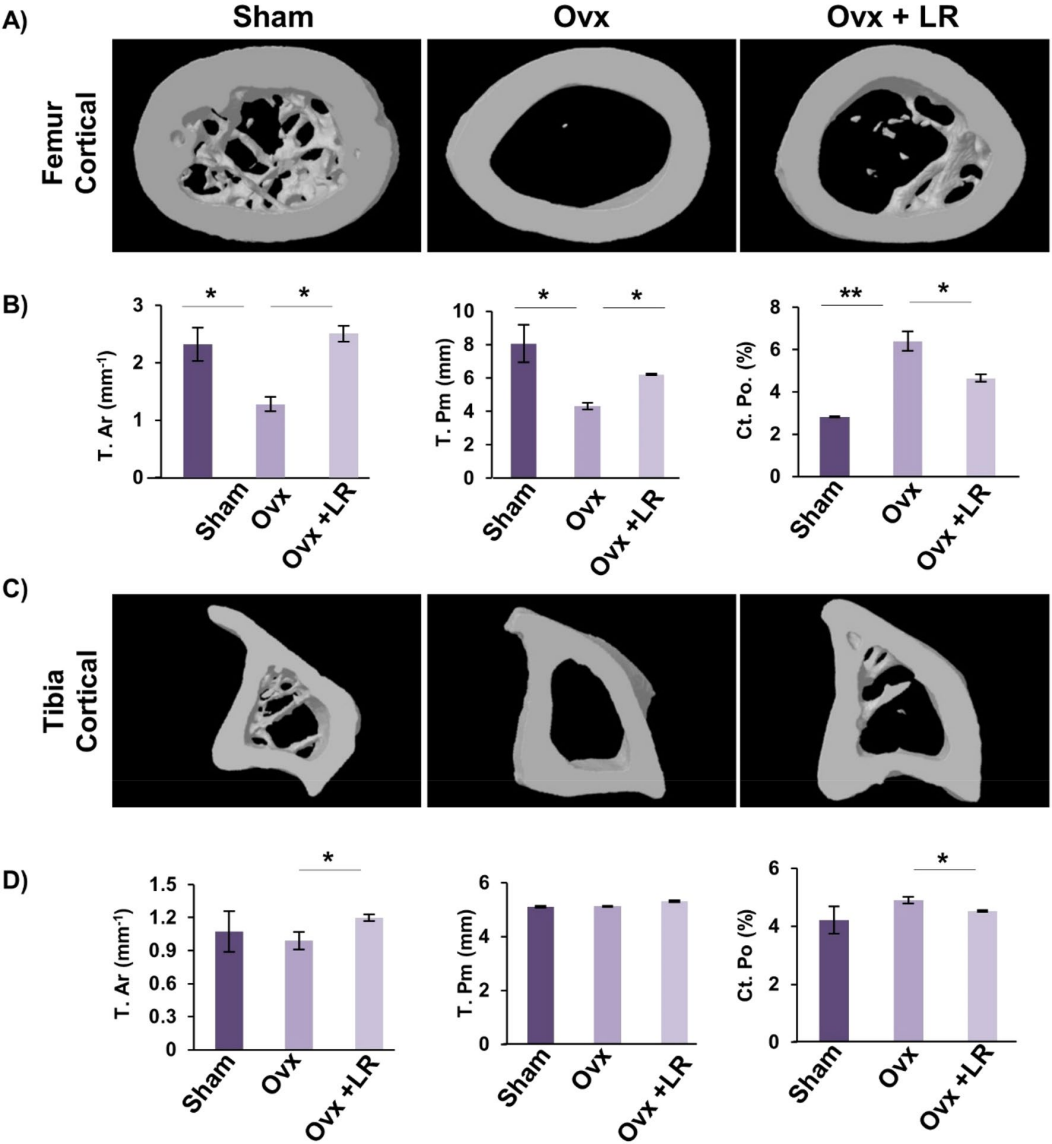
LR administration enhances cortical bone microarchitecture. 3-D u-CT reconstruction of Femur Cortical and Tibia Cortical of all groups. (**A**) Bone micro-architecture of Femur Cortical. (**B**) Histomorphometric parameters of Femur Cortical. (**C**) Bone micro-architecture of Tibia Cortical. (**D**) Histomorphometric parameters of Tibia Cortical. T. Ar., bone tissue area; T. Pm., total cross-sectional perimeter; Ct. Po., cortical porosity. The results were evaluated by ANOVA with subsequent comparisons by Student t-test for paired or nonpaired data. Values are reported as mean ± SEM. The above graphical representations are indicative of one independent experiment and similar results were obtained in two different independent experiments with n = 6. Statistical significance was considered as *p* ≤ 0.05 (*p ≤ 0.05, **p ≤ 0.01, ***p ≤ 0.001) with respect to indicated mice groups.

Moving ahead in our study we were subsequently interested in deciphering the effect of LR-administration on bone histomorphometric parameters. We thus performed high resolution micro-computed tomography (μ-CT) (a gold standard for determining bone-health) for evaluation of bone morphology and quantifying various bone morphometric indices related to bone loss and bone mass. Lumbar vertebrae-5 (LV-5) is considered as one of the most peculiar regions to diagnose early bone loss or osteoporosis^13,14,29^. Thus, we analysed the effect of LR administration on LV-5 trabecular region. Interestingly, μ-CT data clearly pointed towards a significant improvement in LV-5 bone micro-architecture in LR administered group (Fig. 5A). In addition to the micro-architecture, LR administration also significantly increased LV-5 trabecular parameters viz. bone volume per tissue volume (BV/TV) (*p* < 0.05) and trabecular thickness (Tb.Th) (*p* < 0.05) and significantly reduced trabecular separation (Tb.Sp) (*p* < 0.05) (Fig. 5B). Next, we also performed µ-CT analysis of femoral and tibial bones by analysing the effect of LR-administration on various trabecular and cortical indices in mice. Thus, µ-CT was further used to separately quantify various bone indices parameters in femur and tibia bones. In comparison to Ovx group, 3D-micro-architecture images of trabecular region of respective bones showed significant improvement in LR treated group (Fig. 5C). Moreover, during measurement of various indices for femoral and tibial trabecular region, it was found that administration of LR significantly enhanced bone micro-architecture by augmenting the BV/TV ratio (*p* < 0.05), Tb.Th (*p* < 0.05) along with reducing Tb.Sp (*p* < 0.01) (Fig. 5D–F) in Ovx group. Notably, we also found comparable data in cortical region of femoral and tibial bones with improvement in bone micro-architecture along with significant enhancement in bone histomorphometric parameters (Fig. 6A–D). Altogether, our data establishes that LR-administration in Ovx mice improves both trabecular and cortical bone microarchitecture of LV-5, femoral and tibial bones.

### LR elevates both bone mineral density (BMD) and heterogeneity of bones

**Figure 7:**
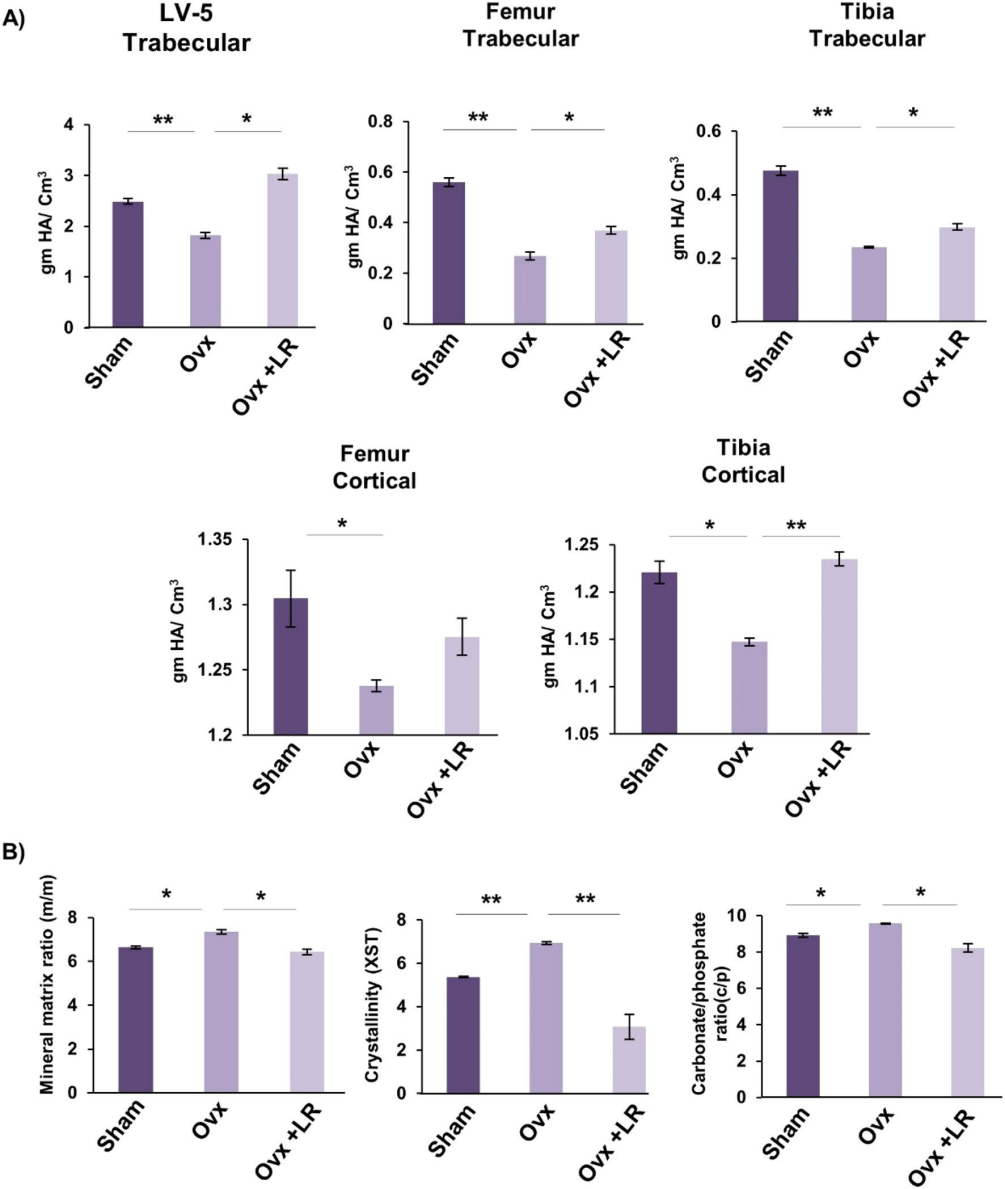
LR administration enhances bone mineral density and heterogeneity of bones. (**A**) Graphical representation of BMD of LV-5, trabecular and cortical regions of femur and tibial bones of all groups. (**B**) Graphical representation of compositional changes in bones as detected by FTIR (bone mineral/organic matrix ratio (m/m), crystallinity (XST) and carbonate to phosphate ratio (C/P). Data are reported as mean ± SEM. Similar results were obtained in two independent experiments with n = 6. Statistical significance of each parameter was assessed by ANOVA followed by paired group comparison. *p < 0.05, **p < 0.01, ***p < 0.001 compared with indicated groups.

To further support our µCT data it was also important to assess the bones for BMD which draws a clear picture about the presence of minerals and their concentration in respective bones. Notably, we observed that administration of LR significantly enhanced the BMDs of both trabecular and cortical regions of LV-5, femoral and tibial bones in Ovx mice (Fig. 7A). Bones have a heterogeneous composition and any loss in heterogeneity has been linked with enhanced fracture risk^30^. Thus, we subsequently performed Fourier transform infrared spectroscopy (FTIR) to evaluate the effect of LR-administration on heterogeneity of bones. The analysis of bone samples revealed that Ovx mice administered with LR had significantly enhanced heterogeneity parameters viz. crystallinity (XST) (*p* < 0.01), carbon content (c/p) (*p* < 0.05) and mineral to organic matrix ratio (m/m) (*p* < 0.05) with respect to Ovx group (Fig. 7B). Taken together these data clearly support and validate our hypothesis that LR-administration not only enhances the BMDs of bones but also preserves their natural heterogeneity, thereby making LR a suitable therapeutic option in the management and treatment of postmenopausal osteoporosis.

### LR enhances bone health via modulating Treg-Th17 cell balance

**Figure 8:**
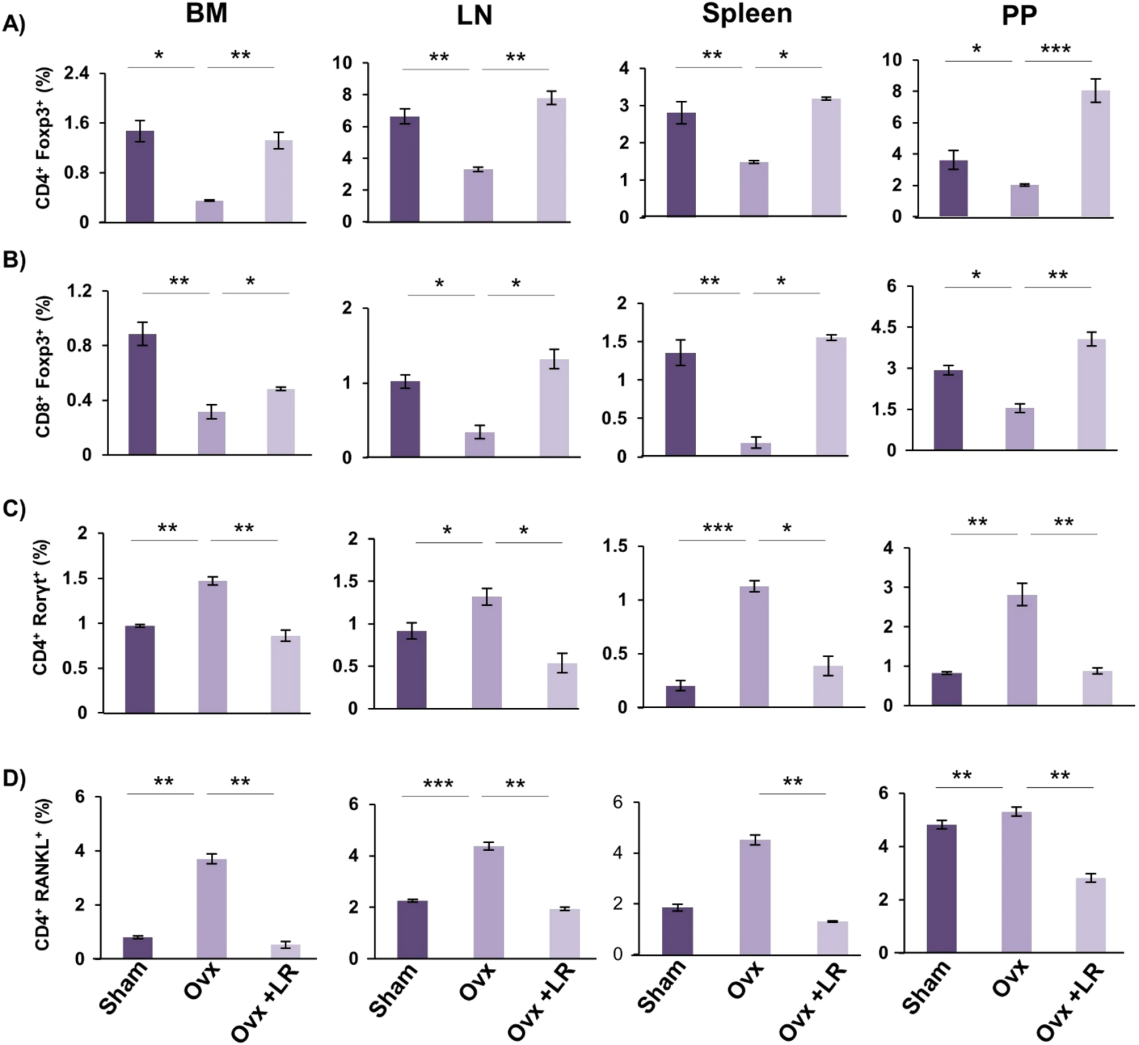
LR intake modulates Treg-Th17 cell balance in vivo. Cells from BM, PP, spleen and LN of mice from Sham, Ovx and Ovx+LR groups were harvested and analysed by Flow cytometry for percentage of (**A**) CD4^+^Foxp3^+^Tregs. (**B**) CD8^+^Foxp3^+^Tregs. (**C**) CD4^+^Roryt^+^Th17 cells. (**D**) CD4^+^RANKL^+^T cells. Data are reported as Mean ± SEM. Similar results were obtained in two independent experiments with n = 6. Statistical significance of each parameter was assessed by ANOVA followed by paired group comparison. *p < 0.05, **p < 0.01, ***p < 0.001, ****p < 0.0001 compared with indicated groups.

Since the role of Treg-Th17 cells is well established in bone homeostasis^10^ thereby we next explored the role of LR in modulating Treg-Th17 cell balance. In this context, we analysed the percentages of CD4^+^Foxp3^+^Tregs, CD8^+^Foxp3^+^Tregs and CD4^+^Roryt^+^Th17 immune cells at various immunological sites such as BM, spleen, PP and LN by flow cytometry. In comparison to Ovx group, LR-administration in Ovx mice led to threefold enhancement in CD4^+^Foxp3^+^Treg cells in BM (Fig. 8A). Interestingly, CD8^+^Foxp3^+^Tregs were also found to be significantly enhanced (*p* < 0.05) (Fig. 8B) along with 1.5-fold reduction in CD4^+^Roryt^+^Th17 cells (Fig. 8C) in LR treated group as compared to Ovx mice (Fig. 8). In spleen, percentage of CD4^+^Foxp3^+^Treg cells were doubled in LR treated group. Strikingly, CD8^+^Foxp3^+^Tregs were also significantly enhanced (*p* < 0.05) along with 1.5-fold reduction in CD4^+^Roryt^+^Th17 cells in LR treated group in comparison to Ovx group. Similarly, significant effects of LR were also observed in LN where the percentages of CD4^+^Foxp3^+^Tregs were doubled (*p* < 0.01), CD8^+^Foxp3^+^Tregs were threefold enhanced (*p* < 0.05) along with simultaneous 1.5-fold reduction in CD4^+^Roryt^+^Th17 cells (*p* < 0.05) in Ovx mice (Fig. 8). Interestingly, PP also had similar trends with a threefold significant enhancement in CD4^+^Foxp3^+^Tregs (*p* < 0.001), 1.5-fold enhancement in CD8^+^Foxp3^+^Tregs (*p* < 0.01) and 1.5-fold significant reduction in CD4^+^Roryt^+^Th17 cells (*p* < 0.01) (Fig. 8) in Ovx mice after LR-administration. Altogether, these results indicate that LR supplementation enhances Tregs population along with simultaneously reducing Th17 cells.

In accordance with earlier reported studies, activated CD4^+^T cells are also known to regulate osteoclast activation by expressing RANKL on their surfaces^31^ and our results too confirmed that oral supplementation of LR significantly downregulated the expression of RANKL on CD4^+^T cells (*p* < 0.01) (Fig. 8 D) in BM, spleen, LN and PP. Overall, these results demonstrate that upon treatment with LR the proportion of anti-osteoclastogenic T lymphocytes (Treg cells) were significantly enhanced. On the other hand, osteoclastogenic T lymphocytes (Th17 cells) were significantly reduced, thereby establishing the role of LR in modulating Treg-Th17 cell balance in osteoporosis.

### LR modulates Treg-Th17 cell differentiation in vitro

**Figure 9:**
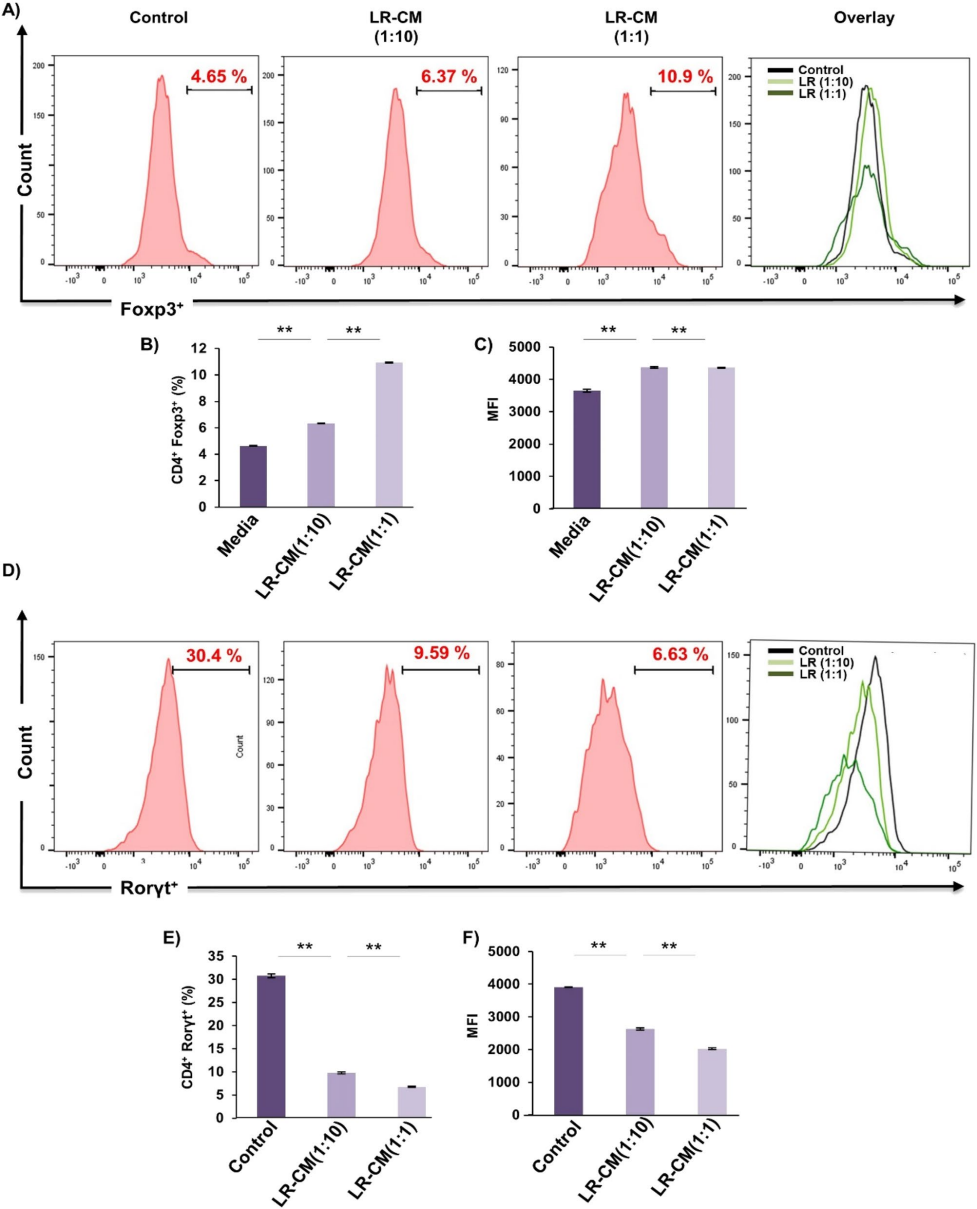
LR modulates Treg and Th17 cell differentiation in vitro. (**A**) For Tregs differentiation, splenic naïve CD4^+^ T cells stimulated with anti-CD3 and anti-CD28 mAbs were incubated with TGF-β1 and IL-2 with or without LR-CM. After 4 days, cells were analysed for Foxp3 expression by FACS. (**B**) Average percentage of CD4^+^Foxp3^+^ cells from two independent experiments of A. (**C**) MFI showing the expression of Foxp3 in CD4^+^ T cells. (**D**) For Th17 differentiation, splenic naïve CD4^+^ T cells stimulated with anti-CD3 and anti-CD28 mAbs, incubated with TGF-β1, IL-6, IL-23 with or without LR-CM. (**E**) Average percentage of CD4^+^Roryt^+^ cells from three independent experiments of D. (**F**) MFI showing the expression of Roryt in CD4^+^ T cells. Data is reported as Mean ± SEM. Similar results were obtained in two independent experiments. MFI, mean fluorescence intensity. Statistical significance of each parameter was assessed by ANOVA followed by paired group comparison. *p < 0.05, **p < 0.01, ***p < 0.001, ****p < 0.0001 compared with indicated groups.

To rule out the possibility of the observed skewed Treg-Th17 cell balance (in our above in vivo results) from other variables we next determined the direct effect of LR treatment on Treg and Th17 cell differentiation in vitro. For Treg cell differentiation, naïve T cells were stimulated with anti-CD3, anti-CD28, IL-2 and TGF-β1 in the presence or absence of conditioned media of LR (LR-CM) at different ratios (1:10 and 1:1). For Th17 cell differentiation naïve T cells were stimulated with anti-CD3, anti-CD28, IL-6, IL-23 and TGF-β1 in the presence or absence of LR-CM at different ratios (1:10 and 1:1). At day 4, cells were harvested and analysed for either CD4^+^Foxp3^+^Treg cells or CD4^+^Rorγt^+^Th17 cells with the help of flow cytometry. Remarkably, we observed that treatment with LR-CM significantly enhanced the percentage of CD4^+^Foxp3^+^Treg cells (1.5-fold with respect to control group) (Fig. 9A–C) along with inhibiting the differentiation of CD4^+^Rorγt^+^Th17 cells (fourfold with respect to control group) in a dose dependent manner (Fig. 9D–F). These results thereby further attest and establish the immunomodulatory role of LR in modulating Treg-Th17 cell balance.

### LR administration skews the expression of cytokines in Ovx mice

**Figure 10:**
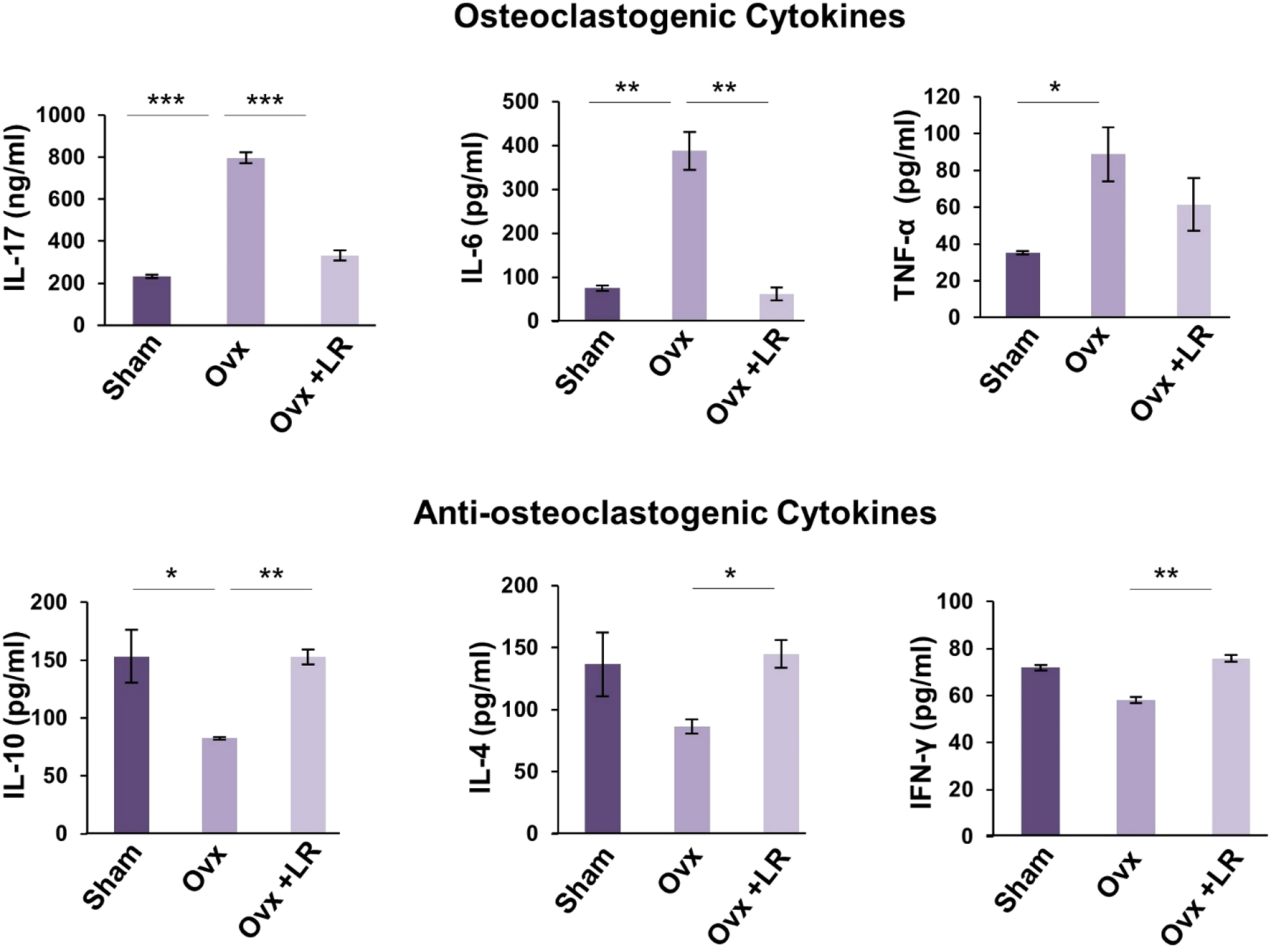
LR skews cytokines balance in Ovx mice. Osteoclastogenic and anti-osteoclastogenic cytokines were analysed in serum samples of mice by ELISA/CBA. The results were evaluated by using ANOVA with subsequent comparisons by Student t-test for paired or non-paired data, as appropriate. Values are expressed as mean ± SEM (n = 6) and similar results were obtained in two independent experiments. Statistical significance was defined as *p* ≤ 0.05, *p ≤ 0.05, **p < 0.01 ***p ≤ 0.001 with respect to indicated mice group.

Cytokines play a key role in the generation and differentiation of osteoclasts viz. pro-inflammatory cytokines (IL-6, IL-17 and TNF- α) which enhances osteoclastogenesis and anti-inflammatory cytokines (IL-4, IL-10 and IFN-γ) which inhibit osteoclastogenesis or enhance bone formation^3,14^. Thus, we next examined the levels of these cytokines in blood serum of mice. Cytokine analysis of blood serum revealed that Ovx mice administered with LR had significantly decreased levels of osteoclastogenic cytokines IL-6 (*p* < 0.01), IL-17 (*p* < 0.001) and TNF-α along with significant augmentation of anti-osteoclastogenic cytokines IL-10 (*p* < 0.01), IL-4 (*p* < 0.05) and IFN-γ (*p* < 0.01) (Fig. 10) as compared to Ovx mice group. Taken together our data strongly supports that LR-administration in Ovx mice leads to enhanced bone health via skewing the cytokine balance. In summary, our findings indicate a crucial role of LR in both bone metabolism and immune responses in attenuating bone loss in postmenopausal osteoporotic condition.

## Discussion

According to International Osteoporosis Foundation (IOF), one-third of the female and one-fifth of the male population across the globe will suffer from osteoporosis related fractures once in their lifetime^32^. Currently it has been estimated that 200 million people worldwide are suffering from osteoporosis^33^. Most of the therapeutic agents that are being currently employed for treating osteoporosis are not only too costly to provide benefits but are also associated with adverse health effects in the long run^6^. Thus, it is necessary to identify entities with no or minimal side effects that can substitute currently available drugs.

In recent years, growing evidences from both murine and human studies have highlighted the beneficial effects of probiotics (*Lactobacillus reuteri, Lactobacillus acidophilus, Lactobacillus casei, Bacillus clausii, Lactobacillus rhamnosus-*GG) in treating various disease conditions^13,14,34–38^. Recently, a study reported by Tyagi et al.^12^ showed that administration of *Lactobacillus rhamnosus* enhanced bone mass in eugonadic mice. We too were interested in studying the impact of LR on bone health under both in vitro and in vivo conditions in Ovx-induced post-menopausal osteoporotic mice model. Strikingly, both our in vitro and in vivo data correlated with previously reported results of Tyagi et al. Our in vitro data clearly indicated that LR-conditioned media exhibits the potential of suppressing RANKL induced osteoclastogenesis in mouse bone marrow macrophages. Also, it exhibits the potential of inhibiting F-actin ring formation in osteoclasts; a key phenotype of mature osteoclasts responsible for bone resorptive activities. These findings of ours clearly establish the direct role of LR in modulating bone health via inhibition of osteoclastogenesis. Furthermore, our in vivo results from SEM and AFM proved that LR-administration inhibits bone loss in Ovx mice. μ-CT analysis further reconfirmed that administration of LR significantly attenuated bone loss by maintaining the bone micro-architecture of LV-5, femoral and tibial bones.

BMD is an important parameter for assessing fracture prevalence in bones^39^. BMD values narrates the risk of bone breakage or development of fracture, as higher BMD signifies lesser risk of fracture whereas lower BMD value relates to higher risk of bone breakage and fracture development^39^. Notably, our experimental outcomes evidently establish that LR has the potential of significantly enhancing bone health by maintaining BMDs of LV-5, femoral and tibial bones. In 2016, Boskey et al.^30^ reported that loss of bone heterogeneity is associated with enhanced brittleness of bone that in turn determines the prevalence of fracture risk. Of note, the physiological heterogeneity of bones should be maintained for their proper functioning^30^. Our results for the first-time report enhancement in heterogeneity of bone samples in LR treated group in comparison to Ovx mice. These data clearly suggest that LR modulates bone health without compromising the heterogeneity of bones thereby confirming LR as a better therapeutic option than the most of currently available anti-osteoporotic drugs (eg. bisphosphonates) that compromise bone heterogeneity (i.e. enhanced homogeneity)^40^ and thus enhance fracture risk in long run.

**Figure 11:**
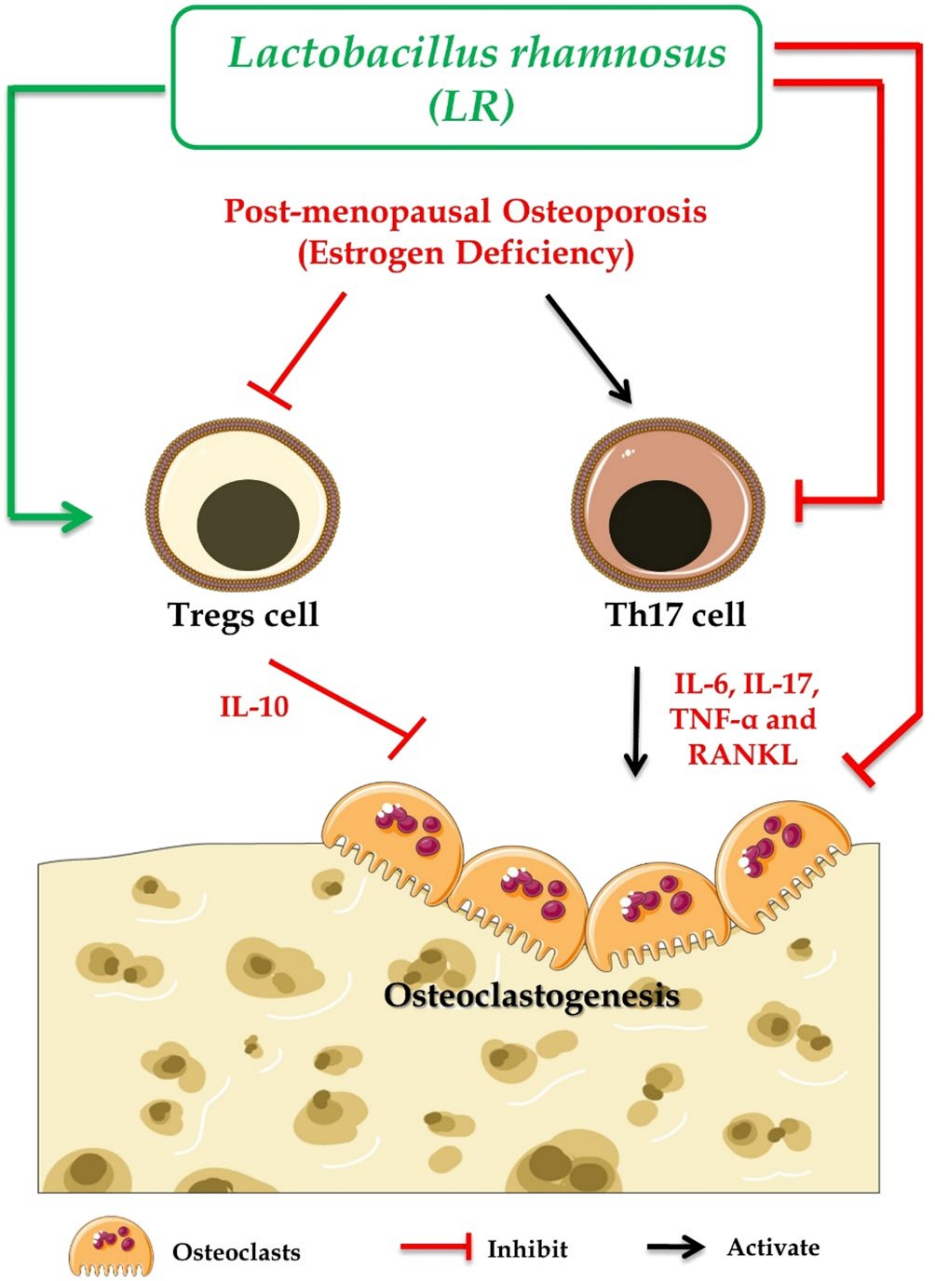
Summary of our results: LR administration attenuates bone loss via inhibiting osteoclasts and modulating the Treg-Th17 cell balance under both in vitro and in vivo conditions. (Image illustrated using Medical Art https://smart.servier.com/).

Although our in vitro data clearly suggested the direct osteoprotective role of LR but we cannot exclude the fact that probiotics exhibits immunomodulatory properties. Also, previous studies from our group has shown that treatment with probiotic *Bacillus clausii*^13^ and *Lactobacillus acidophilus*^14^ enhanced the femoral and tibial bone micro-architecture, bone mineral content via maintaining the homeostatic Tregs and Th17 cell balance in Ovx mice. Study by Tyagi et al.^12^ reported that LR regulates bone mass by stimulating the production of butyrate in mice but unfortunately the immunomodulatory potential (specifically the Treg-Th17 cell axis) of this probiotic was not reported. Building upon these previous evidences, in the present study we elucidated the immunomodulatory properties through which LR inhibits bone loss in Ovx mice. Both bone cells and immune cells shares a common niche (i.e. bone-marrow) during their development, an active field of research termed as Osteoimmunology. Among immune cells, Tregs and Th17 lymphocytes are the key players involved in regulating the bone remodelling process. Cytokines derived from these immune cells such as IL-10 and IL-17 have established roles in regulating osteoclastogenesis^3,40–43^. In this context, we too studied the effect of probiotic LR on Tregs-Th17 cells in BM (prime site of osteoclastogenesis), spleen, PP, LN and our data clearly suggest that LR exerts systemic bone effects via enhancing Tregs population along with simultaneous reduction in Th17 cells. These results were further supported by our serum cytokine data where we observed significant enhancement of anti-osteoclastogenic cytokines such as IL-4, IL-10 and IFN-γ^10^ and down regulation of osteoclastogenic cytokines such as IL-6, IL-17 and TNF-α. Since, our in vitro results clearly pointed to the inhibitory role of LR-CM on RANKL induced osteoclastogenesis we thus investigated the source of RANKL in vivo. One of the major immunological source of RANKL are T cells^31^. Thus, we determined the expression of RANKL on T cells in various lymphoid tissues viz. BM, Spleen, PP and LN. Surprisingly, we found that LR-administration significantly inhibited the expression of RANKL on T cells. These in vivo results are thus in concurrence with our in vitro results wherein LR-CM inhibited RANKL induced osteoclastogenesis. From these data, we observed that LR significantly enhanced the percentage of Tregs along with simultaneously reducing Th17 cells. These results of ours thus clearly establish both direct and indirect link between “LR-Bone-T cells”. In summary, our data clearly establish that LR enhances bone health via maintaining the homeostatic balance between Tregs-Th17 cells (Fig. 11).

Our present study thus for the first time demonstrates the beneficial effects of LR on skeletal health by maintaining the BMD, along with preserving bone micro-architecture and heterogeneity of bones in bilaterally induced ovariectomized mice model via modulating the homeostatic balance of Treg-Th17 cell axis in the host. The present study thus highlights the potential of probiotic LR to be used as a novel osteoprotective agent in the treatment and management of bone related diseases including osteoporosis.

## Materials and methods

### Reagents and antibodies

The following antibodies/kits were procured from eBiosciences (USA): APC Anti-Mouse/Rat-Foxp3 (FJK-16s) (17-5773), PE Anti-Human/Mouse-Rorγt (AFKJS-9) (12-6988), Foxp3/Transcription factor staining buffer (0-5523-00) and RBC lysis buffer (00-4300-54). PE/Cy7 Anti-Mouse CD8 (53-6.7) (100721), PE Anti-Mouse CD254-(TRANCE-RANKL) (IK22/5) (510005). Anti-IFN-γ (clone: XMG1.2), Anti-IL-4 (clone: 11B11), Anti-CD3e (clone: 145-2C11) and Anti-CD28 (clone: 37.51) antibodies were procured from BioLegend (USA). Following ELISA kits were brought from R&D: Mouse IL-10 (M1000B) and Mouse IL-17 (M1700) Quantikine ELISA kits. The Following ELISA kits and reagents were brought from BD (USA): Mouse IL-6 (OptEIA-555240), Mouse TNF-α (OptEIA-560478). Acid phosphatase, leukocyte (TRAP) kit (387A), 3-(4, 5-dimethylthiazol-2-yl)-2,5-diphenyl tetrazolium bromide (MTT), FITC-Phalloidin (P5282) and DAPI were bought from Sigma (USA). Macrophage-colony stimulating factor (M-CSF) (300-25) and Receptor activator of nuclear factor κB-ligand (sRANKL) (310-01), Human TGF-β1 (AF-100-21C), Murine IL-2 (AF-212-12), Murine IL-6 (AF-216-16), Murine IL-23 (200-23), were procured from PeproTech (USA). MRS broth (GM369) was purchased from HiMedia (India). α-Minimal essential media and RPMI-1640 were purchased from Gibco (USA).

### Animals

All in vivo experiments were performed in 8–10-wks old female BALB/c mice. Mice were maintained under specific pathogen free (SPF) conditions at the animal facility of All India Institute of Medical Sciences (AIIMS), New Delhi, India. Mice were fed sterilized food and autoclaved-drinking-water *ad-libitum*. Mice were exposed to bilateral-ovariectomy (Ovx) and sham surgery after anesthetizing them with ketamine (100–150 mg/kg) and xylazine (5–16 mg/kg) intraperitoneally. Later, operated mice were divided into three groups with 6 mice in each group viz. (1) group A: sham operated; (2) group B: Ovx and (3) group C: Ovx+*Lactobacillus rhamnosus* (LR). *Lactobacillus rhamnosus* UBLR-58 (MTCC 5402) was procured from Unique Biotech Ltd., Hyderabad, India.

After one week post-surgery, LR was administered orally as suspension of 400ul (10^9^ cfu/ml) daily in drinking water to Ovx+LR group for a period of 6wks. At the end of experiment, animals were euthanized by carbon dioxide asphyxiation and blood, bones and lymphoid tissues were harvested for further analysis (Fig. 3A). The body weight of animals was recorded at regular intervals (Day 1, 21 and 45) during the experimental period. All the procedures were performed in accordance with the principles, recommendation and after due approval of the protocols submitted to Institutional Animal Ethics Committee of All India Institute of Medical Sciences (AIIMS), New Delhi, India (71/IAEC-1/2018).

### *Lactobacillus rhamnosus* bacterial culture

*Lactobacillus rhamnosus* UBLR-58 (MTCC 5402) was cultured in DeMan, Rogosa, Sharpe media (MRS, HiMedia) overnight at 37 °C (Shaking off). On the following day, overnight culture was subcultured into fresh MRS media and culture was grown until log phase (OD_600nm_ = 0.4) (shaking off). Cells were harvested and washed with 1× PBS to remove traces of MRS broth and centrifuged at 4000 rpm for 10 min. Further, conditioned media (CM) of *L. rhamnosus* was obtained by resuspending the cells either in α-MEM or RPMI-1640 media and incubated for 3 h at 37 °C (orbital shaking 60 rpm). Supernatant was collected by pelleting out the cells, pH neutralized and filtered with 0.22 μm filter. CM was utilized immediately or stored in − 80 °C for further analysis of both osteoclastogenesis and Treg-Th17 cells differentiation assays.

### Generation and characterization of Osteoclasts

Mouse bone marrow macrophages (BMMs) were harvested from femur and tibiae of 8–10 wks old female BALB/c mice and RBC lysis was performed with 1X RBC lysis buffer. Cells were cultured overnight in T25 flask in endotoxin free α-MEM media supplemented with 10% heat inactivated fetal bovine serum (FBS) and M-CSF (35 ng/ml). On the following day, non-adherent cells were collected and cells were seeded in 96-well plate (50,000/well) in osteoclastogenic medium supplemented with M-CSF (30 ng/ml) and RANKL (100 ng/ml) in the presence or absence of LR-CM at different ratio for 5 days. Media was replenished every 3rd day by removing half media and replacing with fresh media supplemented with factors. For evaluating the generation of mature multinucleated osteoclasts tartrate resistant acid phosphatase (TRAP) staining was performed. At the end of incubation, cells were washed twice with 1× PBS and cells were fixed with fixative solution comprised of citrate, acetone and 3.7% formaldehyde for 10 min at 37 °C. After fixation, cells were stained for TRAP as per the manufacturer’s instructions at 37 °C in dark for 5–15 min. Multinucleated TRAP positive cells with ≥ 3 nuclei were considered as mature osteoclasts. TRAP positive multinucleated cells were further counted and imaged using inverted microscope (ECLIPSE, TS100, Nikon). Area of TRAP positive cells was quantified with the help of Image J software (NIH, USA).

### F-actin ring formation assay

After differentiation of bone marrow derived osteoclasts on glass coverslips in 12 well plate, cells were processed for F-actin polymerization staining. After washing twice with 1× PBS, cells were fixed with 4% paraformaldehyde (PFA) for 20 min and permeabilized with 0.1% Triton X-100 for 5 min at RT. To prevent the non-specific binding of antibodies, cells were blocked with 1% BSA for 30 min. After blocking, cells were stained with FITC-labelled phalloidin for 1 h at RT. Finally, nuclei were stained with DAPI and incubated for 5 min at RT. Subsequently, F-actin ring formation was observed using immunofluorescence microscope (Imager.Z2, Zeiss).

### Cell viability or metabolic activity assay

MTT assay was performed to assess the cell viability as a function of cellular metabolic activity. Briefly, BMMs were seeded in 96 well plate (10, 000 cells/well) and incubated for 24 h in CO_2_ incubator at 37 °C. On the following day, cells were treated with LR-CM at different ratios and further incubated for 48 h. On completion of treatment, MTT was added (5 mg/ml) and plate was incubated in CO_2_ incubator at 37 °C for 4 h. After incubation, formazan crystals were dissolved by adding DMSO. The plate was shaken for 5 s (orbital shaking) and reading was taken at 570 nm on a microplate reader (Synergy H1, BioTek).

### Treg and Th17 cell differentiation assay

For isolation of naïve CD4^+^ T cells, lymphocytes harvested from the spleen of 8 wks old mice were incubated with T cell enrichment cocktail (BD) and untouched negatively selected CD4^+^ T cells (10^6^ cells) were seeded in anti-CD3 (5 ug /ml) and anti-CD28 (2 ug/ml) mAbs coated 48-well plate. For in vitro Treg cell differentiation, cells were cultured in RPMI-1640 media and stimulated with anti-IL-4 (5 ug/ml), anti-IFNγ (5 ug/ml), TGF-β1 (5 ng/ml) and IL-2 (10 ng/ml) in the presence and absence of LR-CM at different ratios. For Th17 cells differentiation, anti-IL-4 (10 ug/ml), anti-IFNγ (10 ug/ml) was used together with TGF-β1 (2 ng/ml), IL-6 (30 ng/ml) and IL-23 (20 ng/ml) with or without LR-CM and incubated for 4 days. At day 4, cells were harvested and subjected to flow cytometry for estimating the percentages of CD4^+^Foxp3^+^Treg and CD4^+^Roryt^+^Th17 cells.

### Flow cytometry

Cells were harvested and stained with antibodies specific for Treg and Th17 cells. For surface marker staining, cells were first incubated with anti-CD4-PerCPcy5.5 and incubated for 30 min in dark on ice. After washing, cells were fixed and permeabilized with 1× fixation-permeabilization-buffer for 30 min on ice in dark. Further, cells were stained with anti-Rorγt-PE and anti-Foxp3-APC for 45 min. After washing cells were acquired on BD LSR II (USA). Flowjo-10 (TreeStar, USA) software was used to analyse the samples and gating strategy was done as per previously defined-protocols^13^.

### Scanning electron microscopy (SEM)

SEM for femur cortical region of bones was done as described previously^13,14,29^. Briefly, bone samples were stored in 1%-Triton-X-100 for 2–3 days and later bone samples were transferred to 1XPBS buffer till the final analysis was performed. After preparation of bone slices, samples were dried under incandescent lamp and sputter coating was performed. Subsequently bones were scanned in Leo 435-VP microscope equipped with digital imaging with 35 mm photography system. SEM images were digitally photographed at 100× magnification to capture the best cortical regions. The SEM images were further analysed by MATLAB software (MathWorks, Natick, MA, USA).

### Atomic force microscopy (AFM)

After drying femur bone samples completely in sterile environment with 60 W lamps for 6 h followed by high vacuum drying. Samples were prepared as per requirement for the machine and analysed by Atomic Force Microscope (INNOVA ICON, Bruker) that works under the Acoustic AC mode. This was assisted by cantilever (NSC 12(c) MikroMasch, Silicon Nitride Tip) and NanoDrive version 8 software. It was set at a constant force of 0.6 N/m with a resonant frequency at 94–136 kHz. Images were recorded at a scan speed of 1.5–2.2 lines/s in air at room temperature. Images were later processed and analysed by using nanoscope analysis software. Further, the 3D AFM images were also analysed by MATLAB software (MathWorks, Natick, MA, USA).

### Micro-computed tomography (µ-CT) measurements

µ-CT scanning and analysis was performed as described previously^13,14,29^ using SkyScan 1076 scanner (Aartselaar, Belgium). Briefly, after positioning all samples at right orientation, scanning was done at 50 kV, 201 mA using 0.5 mm aluminium filter and exposure was set to 590 ms. NRecon software was used for carrying out reconstruction process. For trabecular region analysis, ROI was drawn at a total of 100 slices in secondary spongiosa at 1.5 mm from distal border of growth plates excluding the parts of cortical bone and primary spongiosa. The CTAn software was used for measuring and calculating the micro architectural parameters in bone samples. Various 3D-histomorphometric parameters were obtained such as: BV/TV (Bone volume/Tissue volume), Tb.Th (Trabecular-thickness), Tb.Sp. (Trabecular-separation) etc. The volume of interest of u-CT scans made for trabecular and cortical regions were used to determine the BMD of LV5, femur and tibia. BMD was measured by using hydroxyapatite phantom rods of 4 mm diameter with known BMD (0.25 g/cm^3^ and 0.75 g/cm^3^) as calibrator^13,14,29^.

### Fourier transform infrared (FTIR) spectroscopy

Femur cortical bone samples were stored in 1% Triton X-100 for 24 h before being dried with 60 W lamps for 6 h followed by high vacuum drying. Dried bone samples were crushed in mortar-pestle, thereafter bone samples were mixed with potassium-bromide (KBr) at (1:100) ratio for FTIR-analysis. Further, acquisition was performed by using 8400S-FTIR-(SHIMADZU), with a resolution 4 cm^−1^; scan speed 2.5 kHz and 128 scans. The samples were clearly positioned with a prism made of highly refractive material. Savitzky Golay algorithm was used to nullify background noise for obtaining smooth spectra of all analysed-samples^13,14,29^.

### Enzyme linked immunosorbent assay (ELISA) and cytometric bead array (CBA)

ELISA was performed for quantitative assessment of cytokines viz. IL-4, IL-6, IL-10, IL-17 and TNF-α in blood serum using commercially available kits as per the manufacturer’s instructions. For estimating IFN-γ cytokine in serum, CBA was performed as per the manufacturer’s instructions (BD-Biosciences). Fluorescent signals were read on flow analyser and data analysed by BD FCAP-Array software (BD-Biosciences, USA).

### Statistical analysis

Statistical differences between groups were assessed by using analysis of variance (ANOVA) with subsequent comparisons via student t-test paired or unpaired as appropriate. All the data values are expressed as Mean ± SEM (n = 6). Statistical significance was determined as *p* ≤ 0.05 (*p < 0.05, **p < 0.01, ***p < 0.001, ****p < 0.0001) with respect to indicated group.

### Ethical approval

All applicable institutional and/or national guidelines for the care and use of animals were followed.

## Abbreviations

IFN: Interferon
IL: Interleukin
TNF: Tumor necrosis factor
RANKL: Receptor activator of nuclear factor kappa β ligand
Ovx: Ovariectomized
BMD: Bone mineral density
BM: Bone marrow
PP: Peyer’s patches
LN: Lymph nodes
RA: Rheumatoid arthritis
2D: Two dimension
3D: Three dimension
TGF: Transforming growth factor
IBD: Inflammatory bowel disease
